# Medicine

**Published:** 1913-03

**Authors:** J. Michell Clarke


### Progress of the Medical Sciences.

### MEDICINE.

**Suprarenals and Blood Pressure.** Anrep[1] shows experimentally that stimulation of the splanchnic nerves gives a rise of blood-pressure. The curve of the rise of blood-pressure takes place in two phases; the second phase is the larger, and is accompanied by a simultaneous and marked acceleration of the heart-beat. The extent of this acceleration depends upon the pulse-rate obtaining before stimulation of the

[1] *J. Physiol.*, 1912, xlv. 307.

splanchnics; if this were already fast, the acceleration was much less marked. This second phase is also accompanied by a constriction of all the peripheral blood-vessels. Both effects, on the heart and vessels, are due to an increased output of adrenalin into the blood-stream, and are absent on splanchnic stimulation if the suprarenals have been previously extirpated. The same effects take place if the heart or an organ or the vessels of a limb have been denervated.

Further, during the secondary phase there are an increased tone and augmentation of the heart, which is also due to the action of adrenalin on this organ. The author observes that the splanchnic, being the great vasomotor nerve to the body through which the central nervous system regulates the blood-pressure, every change in nervous or muscular activity is probably associated with corresponding changes in the impulses descending the splanchnic nerves and playing on the vascular system. In all these reactions there is a mixture of effects, some due to the direct influence of nerves on the heart and blood-vessels, others to the action of adrenalin set free by the action of the splanchnic nerves on the suprarenal glands. He concludes that every rise of blood-pressure brought about by the agency of the nervous system thus involves the co-operation of the chemical mechanism represented by the suprarenal glands.

**Local vascular reactions.**—In a second paper Anrep[1] discusses the constriction of the blood-vessels obtained in denervated limbs during the rise of blood-pressure produced by splanchnic stimulation. This was regarded by Bayliss as evidence of the existence of a power of local reaction of the blood-vessels to variations of the internal pressure upon their walls. The author shows that all such effects are in reality due to the production of adrenalin (the secretion of which was increased under the conditions of the experiments) and its passage into the circulation, and that a local form of reaction of the walls of the blood-vessels to changes of tension is as yet unproven. The importance of these researches lies in the evidence of a *chemical* production of changes of blood-pressure, both local and general, and they should be read in connection with Elliott's paper[2] on the secretion of adrenalin which results from stimulation of the splanchnic nerves either electrically or through the ordinary paths of the central nervous system by emotion, etc.

**Hexamethylenamin in the treatment of system infections, and as a prophylatic.**—Crowe[3] believes that hexamethylenamin is valuable both as a therapeutic and a prophylactic agent,

[1] *Ibid.*, p. 318. [2] *J. Physiol.*, 1912, xliv. 374.
[3] *Johns Hopkins Hosp. Bull.*, 1912, xxiii. 255.

and from experimental and clinical observations he advocates its use in systemic infections. It is indicated in infections (1) of the genito-urinary tract and typhoid bacilluria ; (2) of the gall-bladder and bile-ducts ; (3) of the respiratory tract, including the ears and nasal sinuses, acute tonsillitis, and some forms of bronchitis (whether it is of value in pneumonia and pulmonary tuberculosis is doubtful) ; (4) in acute poliomyelitis, epidemic meningitis, and meningeal infections secondary to injuries or infectious processes elsewhere. In the Johns Hopkins Hospital it has been given as a routine treatment in all cases in which there is reason to fear a meningeal infection. In cases of compound fracture of the skull he gives evidence to show that the mortality from secondary infection has been reduced since this measure was adopted. Hexamethylenamin has been given in doses of from 40 to 60 grains during the twenty-four hours preceding operation in forty cases of tumour of the hypophysis. When given either by mouth or rectum it appears in the bile and urine almost simultaneously. If the drug is given in a dose of 75 grains daily the amount in the gall-bladder is sufficient to render the bile an unsuitable medium for the growth of bacteria. In administration 2 to 3 grains of hexamethylenamin are added to every ounce of liquid taken, and in this way 60–100 grains may often be given in the twenty-four hours without the patient's knowledge, and without causing any irritation of the stomach or kidneys. In patients who are very ill it may be given per rectum, 50–100 grains to a litre of normal saline solution, allowed to flow into the bowel a drop at a time. Although as much as from 200–300 grains daily for four or five days were given in some cases, in very few were toxic symptoms produced. 100–125 grains daily were administered to children without ill effects. Pain in micturition and hæmaturia occurred in a very few cases : three of these were cases of severe meningeal infection, to whom the drug was given in very large doses. *Post-mortem* it was found that the hæmaturia was of vesical, and not of renal origin. The drug should be given freely diluted. Some few persons show an idiosyncrasy to it, even in small doses, in the form of gastric or urinary irritation, or of skin eruptions.

**Catarrhal Jaundice, Sporadic and Epidemic.**—Cockayne[1] distinguishes two forms of epidemic jaundice, whilst admitting that this distinction is not recognised by some authorities. The first, generally known as Weil's disease, or infectious jaundice, is the more severe and the rarer. It is probably endemic round the shores of the Mediterranean, and milder epidemics have occurred in France, Italy, Germany, and Russia.

[1] *Quart. J. Med.*, 1912–13, vi. 1.

It is probably due to the ingestion of contaminated water or food, but may be due to some biting insect. Primarily it only affects the lowest classes, and is essentially a filth disease.

The B. proteus fluorescens appears to have been the exciting cause in some cases. After an incubation period of two days there is a sudden onset with rigor and high fever; jaundice appears on the third or fourth day, with enlargement of liver and spleen, and nervous symptoms; and from the seventh to the ninth day a remarkable urinary crisis (polyuria and azoturia) with cutaneous petechial hemorrhages are common; other hemorrhages may occur. The mortality varies from 10 per cent. to 60 per cent. in different outbreaks.

The second form, with which the paper chiefly deals, and which the author calls true epidemic catarrhal jaundice, is a milder disease of almost world-wide distribution, and comprises most recorded epidemics of jaundice. There is strong evidence that, unlike Weil's disease, it is air-borne, and is not spread by food or water. The incubation period is from four to ten days, or longer. It is almost certain that the patient has ceased to be infectious when the jaundice appears. There are two main types: (1) Without constitutional symptoms and often without fever, the first sign of illness being jaundice; and (2) with a definite prodromal period, with fever lasting from four to fourteen days before jaundice appears. In most epidemics both types are seen, but in some one only may be present. In the febrile cases there is occasionally a rigor, fever is not as a rule high, and a high temperature does not always connote a severe attack. The liver is always tender, and generally swollen. The spleen may or may not be enlarged. Relapse may occur, but is uncommon; relapsing cases are much more fatal. Convalescence is often slow, weakness being out of proportion to the severity of the symptoms. Deaths are very unusual.

An interesting point is the association of epidemics of jaundice with outbreaks of enteric fever and influenza, especially with the latter: the author thinks this association is accidental. Rolleston considers some cases of sporadic catarrhal jaundice to be of the same nature as the epidemic form, but only includes the febrile cases. In favour of this is the similarity in geographical range, age, and seasonal incidence. Children form nearly half the cases

Little is known of the pathology. In the few fatal cases examined death has been accidental, or due to some other disease.

The relation to acute yellow atrophy of the liver is important; the author brings forward evidence to show the connection between the two. Perhaps the most striking facts are the close resemblance between the early signs and symptoms in acute yellow atrophy and epidemic jaundice, and that in certain epidemics of jaundice in which pregnant women were attacked some of them died of acute yellow atrophy. In such cases he thinks that the additional strain thrown upon the liver by the specific organism of catarrhal jaundice at a time when it was already weakened by the changes incidental to pregnancy proved fatal, though, acting alone, neither pregnancy nor jaundice would have caused such an untoward result. He thinks that sporadic and epidemic catarrhal jaundice and acute yellow atrophy are due in the great majority of cases to the same cause, a specific organism of unknown nature, and suggests the name of "infective hepatitis" as a more accurate one than catarrhal jaundice.

In a paper on **Epidemic Catarrhal Jaundice**[1] Dr. L. Guthrie described ten cases which occurred at the end of 1911, and drew attention to the following points: (1) That mild epidemics have been especially prevalent in this country during the last three years; (2) that though mild in character hitherto, it is possible that at any time they might become more formidable; (3) the prognosis in any case of jaundice in a child, whether sporadic or epidemic, must be guarded, for there is no means of knowing whether it will run a mild course or terminate in acute yellow atrophy of the liver.

Cockayne and Guthrie both refer to the possible relationship or analogy between mumps and catarrhal jaundice. The metastases which occur in mumps, and the fact that other glands, such as the testis and pancreas, may be affected, with or without the parotid, suggests that the infection may reach the parotid and other glands by the blood-stream. In mumps it is possible that the blocking of Steno's duct may be secondary to parotitis, and in jaundice the obstruction of the bile-duct may be secondary to hepatitis. A nurse in the Great Ormond Street Hospital under Dr. Batten developed mumps on December 1st. Between December 7th and 10th six children in the ward also contracted it. On January 2nd another nurse in the same ward developed catarrhal jaundice, but not mumps. A third nurse had mumps on December 10th, with very severe abdominal pain and vomiting, which Dr. Batten attributed to pancreatitis. He is inclined to think that pancreatitis was the connecting-link between mumps and jaundice in these cases. In the discussion on this paper Dr. Goodall doubted a connection between mumps and epidemic jaundice. He believed that there was a specific fever, of which the chief symptom was jaundice, and that its relationship to Weil's disease was a matter for further investigation.

J. MICHELL CLARKE.

[1] *Proc. Roy. Soc. Med.*, 1912, vi., Sect. Dis. Child, 49.

